# A Novel *GEMIN4* Variant in a Consanguineous Family Leads to Neurodevelopmental Impairment with Severe Microcephaly, Spastic Quadriplegia, Epilepsy, and Cataracts

**DOI:** 10.3390/genes13010092

**Published:** 2021-12-30

**Authors:** Hesham Aldhalaan, Albandary AlBakheet, Sarah AlRuways, Nouf AlMutairi, Maha AlNakiyah, Reema AlGhofaili, Kelly J. Cardona-Londoño, Khalid Omar Alahmadi, Hanan AlQudairy, Maha M. AlRasheed, Dilek Colak, Stefan T. Arold, Namik Kaya

**Affiliations:** 1 Neurosciences Department, King Faisal Specialist Hospital and Research Centre, Riyadh 11211, Saudi Arabia; hdhalaan@kfshrc.edu.sa; 2 Translational Genomic Department, Center for Genomic Medicine, King Faisal Specialist Hospital and Research Centre, Riyadh 11211, Saudi Arabia; abinbakheet@kfshrc.edu.sa (A.A.); 437200590@student.ksu.edu.sa (S.A.); 437202397@student.ksu.edu.sa (N.A.); 437200678@student.ksu.edu.sa (M.A.); 437202730@student.ksu.edu.sa (R.A.); halqudairy@kfshrc.edu.sa (H.A.); 3 Clinical Pharmacy Department, College of Pharmacy, King Saud University, Riyadh 11211, Saudi Arabia; mahalrasheed@KSU.EDU.SA; 4 Division of Biological and Environmental Sciences and Engineering (BESE), Computational Bioscience Research Center (CBRC), King Abdullah University of Science and Technology (KAUST), Thuwal 23955-6900, Saudi Arabia; kelly.cardonalondono@kaust.edu.sa (K.J.C.-L.); stefan.arold@kaust.edu.sa (S.T.A.); 5 Department of Radiology, King Faisal Specialist Hospital and Research Centre, Riyadh 11211, Saudi Arabia; KALAHMADI@kfshrc.edu.sa; 6 Department of Biostatistics, Epidemiology and Scientific Computing, King Faisal Specialist Hospital and Research Centre, Riyadh 11211, Saudi Arabia; dkcolak@gmail.com

**Keywords:** *GEMIN4*, homoallelic, novel pathogenic variant, in *silico* prediction, structural modeling, global developmental delay, pediatric cataract

## Abstract

Pathogenic variants in *GEMIN4* contribute to a hereditary disorder characterized by neurodevelopmental features, microcephaly, cataracts, and renal abnormalities (known as NEDMCR). To date, only two homoallelic variations have been linked to the disease. Moreover, clinical features associated with the variants have not been fully elucidated yet. Here, we identified a novel variant in *GEMIN4* (NM_015721:exon2:c.440A>G:p.His147Arg) in two siblings from a consanguineous Saudi family by using whole exome sequencing followed by Sanger sequence verification. We comprehensively investigated the patients’ clinical features, including brain imaging and electroencephalogram findings, and compared their phenotypic characteristics with those of previously reported cases. In *silico* prediction and structural modeling support that the p.His147Arg variant is pathogenic.

## 1. Introduction

The survival motor neuron (SMN) complex is responsible for the assembly and maturation of the Sm-class of small nuclear ribonucleoproteins (snRNPs), the key components of the spliceosomes [1]. The SMN complex consists of at least nine proteins, one of which is the gem nuclear organelle associated protein 4 (GEMIN4). Immunolocalization experiments showed that GEMIN4 is colocalized with SMN in the cytoplasm and in gems. GEMIN4 has also been detected in the nucleoli, suggesting additional roles in ribosome biogenesis [2].

GEMIN4 and its orthologues are essential molecules for neuromuscular activity and vitality [3] and participate in nuclear receptor binding, microRNA biology [4], and nuclear import of the SMN complex [5,6]. Complete gene disruption of *Gemin4* in mice results in embryonic lethality [4,5]. Similar findings have been reported for *Drosophila melanogaster* ortholog *Glos* [4]. These findings suggest that *GEMIN4* and its orthologs play an essential role in the early development of a wide variety of species.

Recently, pathogenic variants in *GEMIN4* have been associated with neurodevelopmental features, microcephaly, cataract, and renal abnormalities (named NEDMCR, OMIM Phenotype MIM Number: 617913) [7,8]. Previous publications have briefly reported the clinical features of affected individuals. Here, we provide the detailed clinical presentation and molecular findings in two siblings with NEDMCR.

## 2. Materials and Methods

### 2.1. Patients and Ethics

**Figure 1:**
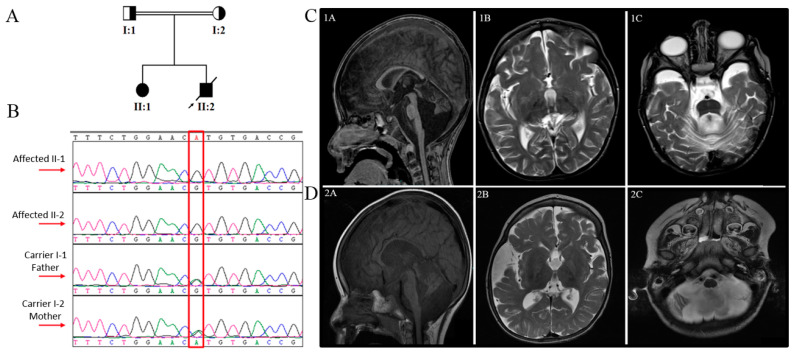
Genetic analysis and brain imaging (MRI) of the patients. (**A**) The pedigree of the family displays two affected individuals. (**B**) Sanger sequencing reveals segregation of the variant in the family members. (**C**,**D**) Brain MRIs of patients with *GEMIN4* novel variant. Sagittal T1WI MRI image (**1A**) showing significant atrophy of cerebellar hemispheres with the dilation of the fourth ventricle communicating with the retrocerebellar cistern secondary to the significant atrophy of the vermis. The corpus callosum shows significant diffuse atrophy. Sagittal T1WI (**2A**) sequence indicates the diffuse atrophy of the corpus callosum. There is significant atrophy of the cerebellum with prominent retrocerebellar cistern and volume loss of the brain stem with prominent prepontine cistern. Axial T2WI sequence (**1B**,**2B**) shows significant dilatation of the ventricular system with prominent extra-axial CSF spaces extending into lateral sulci. There is an asymmetric dilatation of the ventricular system due to periventricular white matter loss (**1C**,**2C**). An incidental prominent basal turn of the cochlea on both sides is observed.

Two siblings from a consanguineous Saudi family (Figure 1A) were recruited to the project. The patients were extensively examined by board-certified pediatric neurologists at the neuroscience clinics at King Faisal Specialist Hospital and Research Center (KFSHRC). Venous blood samples were collected into EDTA tubes from the probands and available family members after obtaining the signed informed consent (approved by the institutional review board, KFSHRC Research Advisory Council, RAC#2120022). Blood was further collected from the affected individuals for culturing Epstein-Barr virus-transformed lymphoblastoid cell lines.

### 2.2. DNA Isolation, PCR, Sanger Sequencing

DNA was extracted from the collected venous blood samples using commercially available kits (Gentra Systems, Minneapolis, MN, USA). The DNA quality and quantity were checked using Nanodrop (ND-1000) or Qubit2.0 Fluorometer (ThermoFisher Scientific Corp., Waltham, MA, USA). Confirmation of the candidate variants obtained from Whole Exome Sequencing (WES) analysis was accomplished using Sanger Sequencing done according to standard protocols.

### 2.3. Whole Exome Sequencing (WES) and Variant Detection

One hundred nanograms of DNA from the index case was utilized as the starting material for the Ion Torrent Proton System. Briefly, DNA was amplified using AmpliSeq kit. The amplicons were then end-repaired and ligated to commercial adapters using DNA ligase. Libraries were barcoded, purified, and the quality assessed according to the manufacturer’s recommendations. Ion proton chips and proton system were used to perform the sequencing (ThermoFisher Scientific Corp.).

### 2.4. In Silico Pathogenicity Prediction Analyses 

In *silico* pathogenicity prediction analyses of the identified variants were performed using multiple prediction algorithms, including SIFT [9], Polyphen-2 [10], ClinPred [11], Fathmm [12], Provean [13], MutPred2 [14], MutationAssessor [15], CADD [16], and MutationTaster [17,18].

### 2.5. Computational Structural Modeling and Analysis of the Novel Variant

GEMIN4 sequence was retrieved from the Uniprot [19]. Residue conservation was assessed with Consurf [20]. Psypred [21], Disopred [22], and P-RaptorX [23] were used to predict secondary structure and disorder. PFAM [24], Interpro [25], and Prosite [26] were employed to identify the domain arrangement. The tertiary protein structure was established with AlphaFold2 [27].

### 2.6. Tandem Mass Spectrometry

Sample preparation, metabolite quantification, use of internal standards, and data acquisition and normalization were as described before [28].

## 3. Results

### 3.1. Clinical Findings

Clinical findings of our patients and the previously reported patients, as well as the variants involved in NEDMCR, are presented in Table 1.

#### 3.1.1. Family A, Patient 1

The patient is an 11-year-old girl with global developmental delay, infantile spasms, epilepsy, and congenital cataract. She was born to a Saudi consanguineous couple after a full-term normal pregnancy via spontaneous vaginal delivery (SVD). At the age of one month, symptoms began as 10-min clusters of psychomotor epilepsy followed by regression and lack of eye contact. She is currently blind and deaf with a drastically delayed function in less than one year. She continued to experience various types of drug-resistant epilepsy ranging from infantile spasm to symptomatic generalized epilepsy, which improved by the age of eight by using levetiracetam and lamotrigine. She eventually stopped taking both drugs at the age of ten. The surgical correction of a congenital cataract was performed at the age of 5 years, with minimal improvement in visual symptoms. She had severe microcephaly and spastic quadriplegia and was unable to control her head or sit independently. At the age of 4.5 years, the head circumference (HC) was 45.5 cm (−3 SD, “Standard Deviation”). At the age of 7 years, it was 46 cm (−4.41 SD). Initial serum amino acid level study revealed a slight elevation in histidine and valine, methionine, and tyrosine; however, subsequent tests revealed a normal profile. A urine amino acid test was unavailable for the patient. Tandem mass spectrometry (MS) revealed a normal result. The basal cisterns were prominent on MRI (Figure 1C) with atrophy of the medulla, pons, cerebellar tonsils, and both cerebellar hemispheres.

#### 3.1.2. Family A, Patient 2

This patient is the younger brother of patient 1. He was born after an uneventful full-term SVD. He suffered from epileptic encephalopathy, infantile spasms, cataracts, and profound global developmental delay. At the age of one month, he developed infantile spasm and up-rolling eyes. He, similar to his sister, continued to have various types of seizures. He suffered from aspiration on a frequent basis, resulting in recurrent pneumonia and the need for nasogastric tube (NGT) feeding. He passed away at the age of nine years secondary to aspiration pneumonia sepsis. On examination, he showed microcephaly, dolichocephaly, and dysmorphic features, with a high arched palate, low set ears, wide depressed nasal bridge, and sialorrhea. His HC was 46 cm (−3.25 SD) at the age of 4.5 years. He had a cataract in his left eye, hypotonia that progressed to spasticity, as well as brisk plus 3 symmetric reflexes. At the age of two years, he developed scoliosis. The EEGs (electroencephalograms) done at the age of one, two, and three years showed hypsarrhythmia with flexor spasm and clinical seizures. An MRI was performed at the age of three years, displaying an enlargement of the subarachnoid spaces surrounding the frontal, temporal, and posterior fossa structures, suggestive of brain underdevelopment with vermian hypoplasia and persistent cavum interpositum (Figure 1D). His urine analysis showed an elevated glycine value of 2192 mmol/mol creatinin, whereas serum amino acid analysis was normal. The patient last presented at the age of five, and was well controlled on valproic acid and clobazam. He lasted two years without a seizure.

### 3.2. Exome Sequencing and In Silico Functional Prediction Analyses

We performed WES to identify plausible disease-causing variant(s). Our analysis, coupled with previously published variant filtering processes [30,31,32,33,34], yielded a novel homozygous missense variant in exon 2 of GEMIN4 (NM_015721: c.440A>G:p.His147Arg) as the only candidate underlying the disease. Then we used Sanger sequencing for the segregation analysis and confirmation of the variant in the family. The analysis revealed that the variant was fully segregated with the phenotype. In other words, the affected individuals were homozygous, whereas the parents were carriers (Figure 1A,B). We also searched for the presence of the variant in various databases such as gnomAD, ExAC, dbSNP, and 1000 Genomes, in addition to the Saudi Human Genome Program, including 2379 in-house Saudi exomes. This pursuit did not point out any positive hit assuring the novelty of the variant. To understand the likely deleterious effect of the variant, we utilized various in *silico* prediction tools and computational structural modeling.

**Figure 2:**
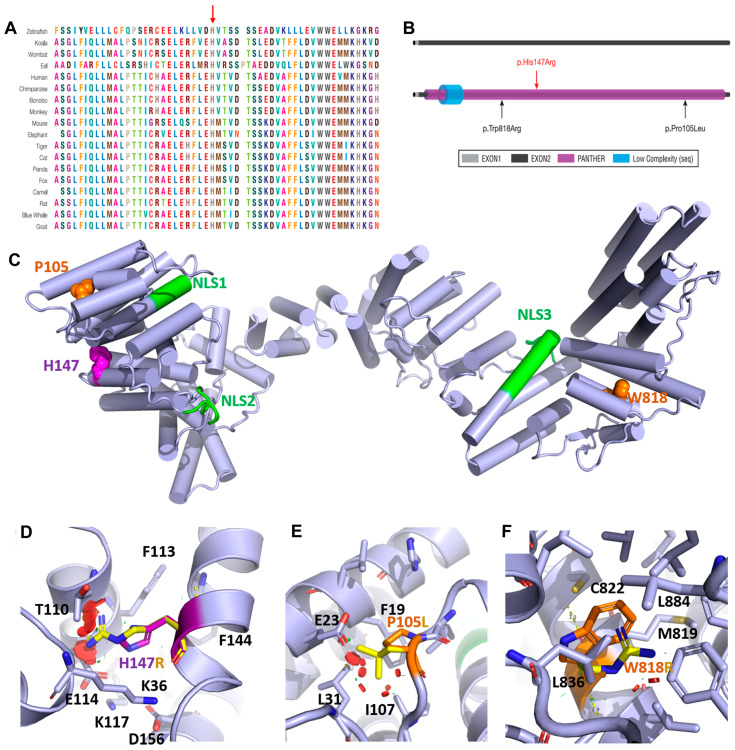
Multiple sequence alignment, schematic drawing of the protein and structural modeling of p.His147Arg. (**A**) The alignment indicates the conservation of the region surrounding the variant site. (**B**) All the reported variants are presented on the schematic drawing of the protein. (**C**) 3D structural models of GEMIN4 predicted with AlphaFold2. Helices are shown as cylinders, and the putative NLS are colored. The residues substituted in the patients described in this report and previous studies are colored in magenta and orange, respectively. (**D–F**) Zoom into the molecular environment of each variant, with the carbon atoms of the wild-type residues and the variants that are colored in yellow. Clashes introduced by the substitution are represented by red circles, where the orientation and diameter show the direction of clashes, and the disc’s thickness illustrates the severity of clashes. Figures were prepared with PyMOL (pymol.org).

In *silico* pathogenicity analyses of the variant using multiple algorithms infered its effect as damaging or disease-causing, including SIFT (damaging, with a score of 0.005), Polyphen-2 (probably damaging, with a score of 0.999), MutationTaster (disease-causing, with score of 1), and others (Supplementary Table S1). We also evaluated the pathogenicity of the previously identified variants causing the disorder. Our evaluation also revealed those as damaging or disease-causing (Suplementary Table S1). The multiple sequence alignment analysis using ClustalW2 for region spanning p. His147Arg indicates the conservation of the mutated residue across species (Figure 2A).

### 3.3. Computational Structural Modeling and Analysis of the Novel Variant

Gemin4 is a 1058 amino acid protein with an estimated molecular mass of 119.9kD [2]. Its 3-dimensional structure is unknown, and database searches did not reveal any recognizable domain; however, the Ensembl database predicts a short low complexity domain with a PANTHER domain (Figure 2A). Bioinformatic analysis pointed to the existence of three classical (pat7 subtype) nuclear localization sequences (NLS), two in the N-terminal half of the protein (NLS1; residues ~62–69 aa and NLS2; residues ~199–206 aa) and one located in the C-terminal half (NLS3; residues ~714–735). Experimental assays deleting the predicted NLS motifs have demonstrated that NLS2 is necessary and sufficient for the nuclear import of cargoes [4]. The function of the other two putative NLS motifs remains unknown. Computational predictions of tertiary and secondary structure features support the notion of Gemin4 being an elongated all-helical protein, reminiscent of protein structures that serve as scaffolds for assembling protein complexes (Figure 2C). Structural modeling by AlphaFold2 [27] places His147 with a high confidence score (91.44) in a helical region in the vicinity of NLS1 and NLS2. In this model, the highly conserved His147 (conservation score = 0.79) has a key role in tethering several helices together through forming hydrogen and cation-pi bonds with Glu114 and Lys36, respectively, while offering hydrophobic contacts for surrounding polar moieties from Thr110, Phe113, and Phe144. The substitution of His147 with a much larger and charged arginine would either produce serious clashes in the structure or leave an unfavorable hole in this structural region, depending on the arginine rotamer that would prevail (Figure 2C). The resulting structural perturbations could hamper the capability of Gemin4 to bind certain ligands, may affect the accessibility of NLS2, and could lead to an unstable and more rapidly degradable protein. This effect is similar to those evoked by previously reported variants (p.Pro105L and p.Trp818R) that are both destabilizing by introducing clashes and gaps in a hydrophobic environment of the protein fold. P105 is in close proximity to His147, whereas Trp818 is located on the opposite site of the structure (Figure 2D−F).

## 4. Discussion

Homozygous pathogenic variants in *GEMIN4* cause the NEDMCR syndrome. Thus far, only two variants, p.Trp818Arg [29] and p.Pro105Leu [7,8], have been reported to cause the disease. This paper highlights a novel homozygous transition (NM_015721:exon2: c.440A>G:p.His147Arg) detected in two siblings born to a consanguineous Saudi family. Clinical examinations revealed similarities and differences between our patients and previously reported individuals. Atypical features such as hearing and speech impairment were found in patient 1 while dolichocephaly, scoliosis, and dysmorphic features were observed in patient 2 who had a high arched palate, low set ears, wide depressed nasal bridge, and sialorrhea. Nonetheless, similarity was seen in the dysmorphic features between patient 2 (c.440A>G:p.H147R) and patient 6 (c.2452T>C:p.W818R) as both had a high arched palate and microcephaly. Recurrent chest infections were noticed in patients 2, 5, and 6, whereas patient 1 exhibited reported NEDMCR syndrome phenotypes. Regarding the renal profile, patient 2 showed an increase in glycine level in urine (tested one time, only). Such increase may be due to valproic acid (VA) since it has been typically associated with hyperglycinuria [35,36]. The MRIs of patients 1 and 2 were almost identical as they showed significant generalized atrophy in the cerebellar hemispheres, corpus callosum, and brain stem. A symmetric dilatation of the ventricular system was also observed due to periventricular white matter loss. However, an enlargement of the subarachnoid spaces was seen in patient 2, suggestive of brain underdevelopment.

The exact relationship between *GEMIN4* pathogenic variants and the syndrome, as well as its potential mediation by SMN complex disruption, are unclear [8]. The observed similarities among the seven patients, collectively presenting three different variants, are consistent (Table 1) [7,8,29]. Previously, three studies have described the effects of this syndrome on patients’ neurological functioning [7,8,29]. Cataracts were the most common phenotypic feature found in the study by Patel et al. (2016) [8]. The same study linked several genes, including *GEMIN4,* to cataracts using developmental lens expression and gene-network analysis [8]. A different study on prescreened multiplex consanguineous families described 33 novel candidate genes [29]. The study reported three unrelated families with three children harboring the same variant in *GEMIN4*. All three patients exhibited typical characteristics of the NEDMCR syndrome, and two of them showed stones and tubulopathy. In addition, they presented with severe dystonia and osteopenia. Maddirevula et al. (2018) [7] reported the same variant identified by Patel et al. (2016) and additional features that could suggest a phenotypic expansion of potentially distinct allelic disorders [8].

In summary, this report presents the full clinical features of two patients with a novel *GEMIN4* pathogenic missense variant (c.440A>G; p.His147Arg) that causes NEDMCR syndrome. Our study provides a detailed comparison of the published cases and reports additional clinical features associated with the disease, such as hearing and speech impairment, dolichocephaly, scoliosis, and dysmorphism, and expands its phenotypic spectrum.

## 5. List of the Databases

https://www.ncbi.nlm.nih.gov/search/
http://asia.ensembl.org/index.html
https://www.genecards.org/
http://genome.ucsc.edu/
https://gnomad.broadinstitute.org/
http://www.hgmd.cf.ac.uk/ac/index.php
https://www.lovd.nl/
https://evs.gs.washington.edu/EVS/
http://www.pantherdb.org

## Figures and Tables

**Table 1:** Clinical and molecular findings of patients in this study, as well as the previously reported variants associated with NEDMCR in the literature.

	This Study		Maddirevula et al., 2019 [7]	Patel et al., 2017 [8]	Alazami et al., 2015 [29]		
	Patient 1	Patient 2	Patient 3	Patient 4	Patient 5	Patient 6	Patient 7
Gender	F	M	F	NA	F	M	F
Age of onset	Few months	1 month	7 year	NA	13 year	1 year	5 year
Consanguinity	Yes	Yes	Yes	NA	Yes	Yes	Yes
Variant (cDNA) *	c.440A>G	c.440A>G	c.314C>T	c.314C>T	c.2452T>C	c.2452T>C	c.2452T>C
Variant (Protein) *	p.His147Arg	p.His147Arg	p.Pro105Leu	p.Pro105Leu	p.Trp818Arg	p.Trp818Arg	p.Trp818Arg
Variant Type	Missense	Missense	Missense	Missense	Missense	Missense	Missense
Seizure	Yes	Yes	Yes	Yes	Yes	Yes	Yes
GDD	Yes	Yes	Yes	Yes	Yes	Yes	Yes
Cataract	Yes	Yes	Yes	Yes	Yes	Yes	Yes
Hearing impairment	Yes	NA	Yes	NA	NA	NA	NA
Microcephaly	Yes	Yes	NA	NA	Yes	Yes	Yes
Dolichocephaly	NA	Yes	NA	NA	NA	NA	NA
Scoliosis	NA	Yes	NA	NA	NA	NA	NA
Dysmorphic features	NA	High arched palate, low set ears, wide depressed nasal bridge, sialorrhea.	NA	NA	Peculiar orientation of alae nasae.	Micrognathia, small mandible, high arched palate, and small bell-shaped thoracic cage. HC = 37 cm (<3rd centile)	No
Motor difficulties	Yes	Yes	Started walking at the age of 2 years	NA	Yes	Yes	Yes
Renal dysfunction	NA	Glycine elevated	NA	NA	Yes	Yes	NA
Chest infections	No	Yes	NA	NA	Yes	Yes	NA
MRI	Medulla, pons, cerebellar vermis, and both cerebellar hemispheres were rather small in size, with enlarged cistern magnum and the box-like appearance of the fourth ventricle.	Generalized atrophy, septum pellucidum was patent and vermian hypoplasia	Small atrophic cerebellum with prominence of the posterior fossa CSF spaces	NA	Bilateral symmetrical alteration of the signal intensity of the white matter of both cerebral hemispheres in the form of bright signal intensity on T2W and flair images, a picture probably reflecting disturbed myelination	Markedly prominent cortical sulci and subarachnoid cisterns were noted	Hypomyelination and callosal thinning

Abbreviations: F: Female/M: Male; GDD: Global developmental delay; MRI: Magnetic resonance imaging; NA: Not available. * Variant naming was based on NM_015721.

## Data Availability

Not applicable.

## Supplementary Materials

The following are available online at https://www.mdpi.com/article/10.3390/genes13010092/s1, Supplementary Table S1: Comprehensive in *silico* pathogenicity prediction analyses of the variant identified in this study, as well as previously identified variants for this disorder, using multiple prediction algorithms.
